# My 40-year Career as a Hematologist – Facing Clinical Challenges and Changing My Research – Focus from Glycolipids to Therapy Resistance –

**DOI:** 10.14789/jmj.JMJ23-0019-R

**Published:** 2023-08-25

**Authors:** MASAAKI NOGUCHI

**Affiliations:** 1 Department of Hematology, Juntendo University Urayasu Hospital, Chiba, Japan; 1 Department of Hematology, Juntendo University Urayasu Hospital, Chiba, Japan

**Keywords:** glycolipid, resident-centered professional society presentation, hematopoietic tumor, mechanism for therapy resistance

## Abstract

It has been about 40 years since I graduated from Juntendo University School of Medicine in 1983. For 5 years after graduation, I was engaged in research on glycolipids in the Biochemistry Unit of the University of Tokyo. Later, I wrote and published papers on glycolipids. Eventually, I began to work here at the Department of Hematology. In 2000, in the 17th year after my graduation, I began to work at the Department of Hematology, Juntendo University Urayasu Hospital, as the only physician stationed there. I had to work long hours, until late night, to manage the many inpat As described in our department's homepage, 145 (64%) of the 227 presentations made at professional society meetings were made by residents. During this period, 15 new residents joined this department. In 2015, contributions by residents were accepted for publication by high-impact-factor journals, such as the Journal of Clinical Oncology. With the support of our Chairman Ogawa, in April 2023, I began to work as a specially appointed professor at the current department. Recently, I have begun to feel deeply grateful for Juntendo university's academic motto of “Benevolence,” its principle of “Uninterrupted Advancing,” and its academic position of “three noes principle, *Sanmu* Principle (no discrimination based on gender, nationality, or academic background)”. I hope that you will all remain active under these principles and ideas, while taking due care of your own health. I wish to express my appreciation for your continued support and cooperation.

## Preface

I am pleased to announce that I will be retiring at the end of March 2023. I would like to express my sincere gratitude to Chairman Hideoki Ogawa and the many others who have guided and supported me throughout the years.

## Before Urayasu Hospital

**Figure 1:**
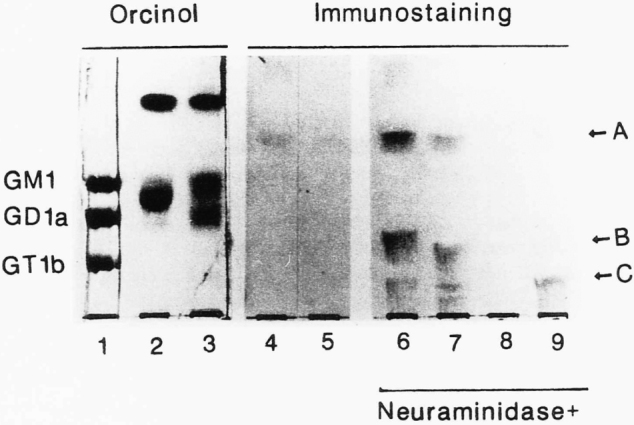
TLC immunostaining of intact and neuramini dase-treated gangliosides from spleens (BALB/c and NZB mice)stained with sera from NZB mice, followed with peroxidase-conjugated goat anti-mouse lgM antibody^1)^ The bands were stained with orcinol-H2S04 reagent (lanes l to 3), and by TLC‐immunostaining without (lanes 4 and 5) and with neuraminidase treatment (lanes 6 to 9) of the TLC plates. Standard gangliosides, GMl, GDla, and GT1b (lane l), gangliosides from fresh weight 100 mg of BALB/c mouse spleens (lanes 3,5 and 9) and NZB mouse spleens (lanes 2,4 and 6), monosialogangliosides (lane 7) and di plus trisialogangliosides (lane 8) from fresh weight 100 mg of NZB mouse spleens were spotted on the plates, respectively. At least, three monosialogangliosides A,B, and C (shown by arrows) from NZB mouse spleens as well as BALB/c mouse spleens were reacted with lgM autoantibodies in sera from NZB mice only after neuraminidase treatment (lanes 6,7, and 9).

**Figure 2:**
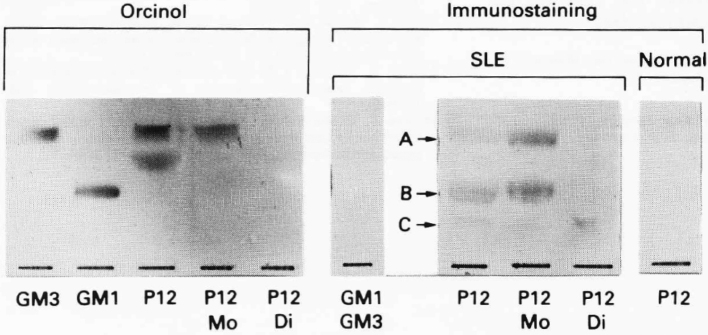
Thin layer chromatography immunostaining of acidic glycosphingolipids^2)^ From P12 cells with serum samples from a patient with SLE and a normal control subject. The acidic glycosphingolipids from P12 cells are abbreviated to P12. The monosialo- and disialo glycosphingolipids from P12 cells abbreviated to P12Mo and P12Di, respectively. The left hand plate was stained with orcino-H2SO4 reagent. The right hand plate was immunostained with a serum sample containing the highest detected titer of IgG antibodies to P12 and a normal control. Monosialogsphingolipids A, B, and C were stained by serum samples from the patient but not the normal control subjects.

**Figure 3:**
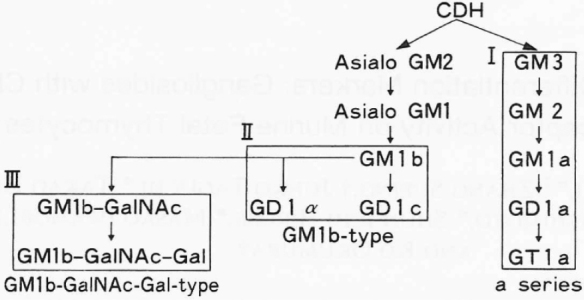
Ganglioside synthetic pathways during T cell developecnt in mice^3)^ Gangliosides are produced by the sequential addition of sugars to ceramide dihexosides (CDH). Three ganglioside synthetic pathways (I, H, and III) are used during T cell development in micc: I, (a series) CDH plus NeuAc or NeuGc (neuramic acid, GM3), GM3 plus N-acctylgalactosamine (GM2), and GM2 plus galactose (GMla); Ⅱ, (GMlb- type). CDH plus NeuAc_acctylgalactosamine (asialo GM2), asialo GM2 plus galactose (asialo GMl), and asialo GM l plus neuramic acid (one; GMlb, two; GMlα, or GMlc and ⅡI, (GMlb-GalNAc-Cal-type).

**Figure 4:**
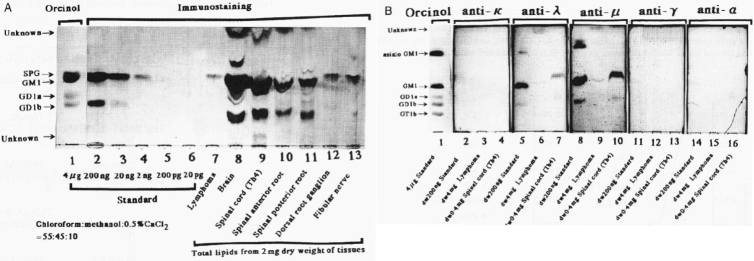
Thin-layer chromatography (TLC) immunostaining of an extract from the patient's neural tissues stained with patient serum IgM λ antibody in the patient's serum preferentially bound glycolipids GM1 and GD1b^4)^. (A)TLC immunostaining with serum followed by anti-IgM antibody (lanes 2-13): lanes 1-6, purified glycolipid mixture as a standard [sialylparagloboside(SPG), GM1, GD1a and GD1b]; lane 7. lipids from the patient's lymphoma cells; lane 8, brain; lane 9, spinal cord (Th4); lane 10, spinal anterior root; lane 11, spinal posterior roots; lane 12, spinal dorsal root ganglion: lane 13, fibular (common peroneal) nerve. (B) TLC immunostaining with serum followed by anti-human immunoglobulin antibodies: anti-κ light chain (lanes 2-4), anti-λ light chain (lanes 5-7), anti- μ heavy chain (lanes 8-10), anti- γ heavy chain(lanes 11-13) and anti-α heavy chain (lanes 14-16).

I graduated in 1983, about 40 years ago, from Juntendo University School of Medicine. During the first 5 years after graduation, I worked at the Department of Collagen Disease and Rheumatology. In those days, close attention was being paid to research on serum phospholipid autoantibodies in patients with systemic lupus erythematosus (SLE). Under such circumstances, I began to engage in research on glycolipids under the guidance of Associate Professor Iwamori (Second Department of Biochemistry, School of Medicine, University of Tokyo). My research was later focused on antigen epitopes recognizing autoantibodies to glycolipids rather than phospholipid autoantibodies, and I published several papers (Figures 1-4). All of these studies were based on analyses using thin-layer chromatography and immunostaining.

Figure. 1 shows the antigen-recognizing site of autoantibodies from NZB mice (a model of SLE being studied by Prof. Shirai, Second Department of Pathology) explored by immunostaining on thin-layer chromatograms. Orcinol staining indicates the control position. Neuraminidase is an enzyme that digests the sialic acid at the sugar chain terminal^1)^.

In 1990, I received my PhD degree in medicine. My doctoral thesis was entitled “Analysis of Anti-lymphocyte Antibodies and Corresponding Glycolipid Antigens in Blood of Patients with Systemic Lupus Erythematosus by Means of TB Cell Lines and Cellular Enzyme Antibody Technique.” As shown in Figure 2, an anti-lymphocyte antibody was detected in the blood of patients with SLE, and the antigen-recognizing site of this antibody was shown to be glycolipids, including at least 3 types of sialic acid. The presence of these glycolipids, accompanied by hypocomplementemia, seemed to be responsible for the lymphopenia in SLE^2)^.

Figure 3 and Table 1 illustrates that in mouse fetuses, the thymus lymphocytes show gradual extension of the sugar chain of the cell surface glycolipids, as these lymphocytes undergo differentiation during the course of intrauterine development^3)^.

**Table 1:** Summary of T Cell Surface Markers during Ontogency in Mice^3)^

	Days of gestation			
Surface markers	13-14	14-15	15-17	17 days to 4 weeks
GM1a (a series)	±	＋	±	−
GMlb-type	±	＋	＋	−
GM1b-GalNAc-Gal	−	−	±	＋
Asialo GMI	−	±	±	±
Thy-1	±	＋	＋	±
TCR (γδ)	±	＋	−	−
IL-2R	−	＋	−	−
CD4	−	−	＋	±
CD8	−	−	＋	±
TCR (αβ)	−	−	±	＋

*Note*. +, increased; ±, slightly changed; -, decreased.

Figure 4 illustrates that the anti-GM1 and anti-GD1b antibodies in the blood of a patient with B-cell lymphoma induced multiple peripheral neuritis through reactions with the peripheral nerve myelin sheath. This seemed to play a significant etiological role^4)^.

To expand my clinical knowledge and skills, I also worked at the Tokyo Metropolitan Matsuzawa Hospital, the Koshigaya Municipal Hospital, etc. Then, I began to work at the Department of Hematology. Moving from such departments to the department of hematology seems to be a rare career choice for physicians. I also worked at the departments of hematology, etc., at the Shizuoka Hospital, Kameda Medical Center, etc., for the purpose of clinical training.

## After Urayasu Hospital

In 2000, in the 17^th^ year after my graduation, I began to work at the Department of Hematology, Juntendo University Urayasu Hospital, as the only physician stationed there. The number of patients visiting my office increased gradually, and I became rather busy. Before long, the number of inpatients at this department increased to about 30, and I had to work long hours, until late night, to manage the many inpatients as well as outpatients. I was often called at night and also on weekends or holidays, including while I was bathing or asleep, to manage sudden changes in the conditions of patients. After about two years, another physician was sent to this department as a rotation staff member from the medical office. In 2007, I was appointed as a Senior Clinical Associate Professor at the Department of Hematology, Juntendo University Urayasu Hospital. The number of physicians working at this department then increased gradually, and many residents and physicians working here began actively committing themselves to making presentations at professional society meetings and writing papers in English for publication.

**Figure 5:**
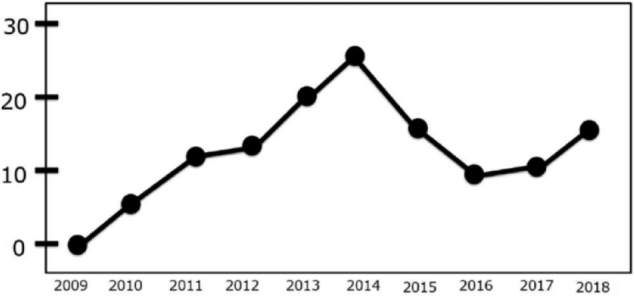
Annual conference presentations by Urayasu Hospital hematology interns Home page URL: https://www.hosp-urayasu.juntendo.ac.jp/medicalcare/hematology/^5)^

In 2013, I was appointed as Professor at the Department of Hematology, Juntendo Urayasu Hospital. As described in our department's homepage which we further enriched, 145 (64%) of the 227 presentations made at professional society meetings were by residents. During this period, 15 new residents joined our department. Figure 5 shows the annual number of presentations made at professional society meetings by the residents of Urayasu Hospital. The number is large and I served as a guide to many of these residents when they made presentations of diverse cases^5)^.

**Figure 6:**
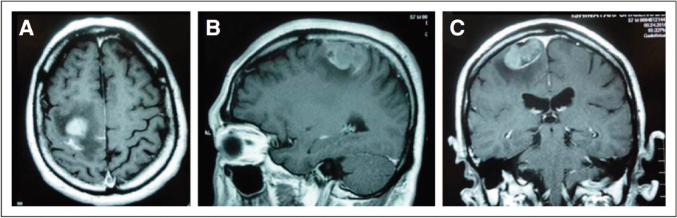
A brain magnetic resonance imaging examination (T1-weighted image) revealed a tumorous lesion (30 x 30 mm) showing a homogeneous and intense gadolinium enhancement in the right frontal to parietal lobe, surrounded by edema. Bleeding inside the tumor was also(Figure 6A, noted, axial; Figure 6B, sagittal; Figure 6C, coronal)^6)^

Figure 6 and Table 2 shows a very rare case encountered at our hospital. The paper on this case was accepted for publication in the Journal of Clinical Oncology, which has a high-impact-factor.

**Table 2:** Summary of primary CNS mantle-cell lymphomas in the literature^6)^

Patient	Age (Years)	Sex	Location	Markers	IPI	PCNSL IPI	Treatment	Outcome	Reference
1	74	F	Frontoparietal region/CSF	CD5^+^ CD20^+^ CD10^−^ CD23^−^	LI*	High	MTX	Dead, 1 month	4
2	29	M	CSF	CD5^+^ CD20^+^ lgG^+^	Low*	Low*	ASCT	CR	5
3	55	M	Frontal lobe/CSF	CD5^+^ CD20^+^ CD10^+^ CCND1^+^	Low	Low	MTX,Ara-C	Dead, 4 months	Current article

Abbreviations: Ara-C, cytarabine; ASCT, autologous stem-cell transplantation; CCNDl, cyclin D1;CR, complete remission; lgG, immunoglobulin G; IPI, International Prognostic Index; LI, low intermediate; MTX, methotrexate; PCNSL, primary CNS lymphoma. *Concerning.

This was a case of double hit mantle cell lymphoma of the central nervous system^6)^.

**Figure 7:**
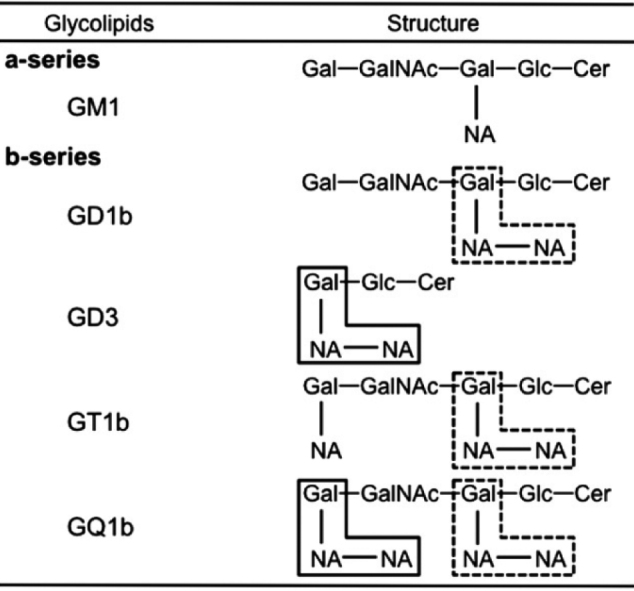
Structures of GM1, GD1b, GD3, GT1b, and GQ1b gangliosides^7)^ Structures of GM1, GD1b, GD3, GT1b, and GQ1b gangliosides. Three terminal residues of NeuNAc(α2-8) NeuNAc(α2-3) Gal in GD3 and GQ1b gangliosides (solid lines) and three internally located terminal residues of NeuNAc(α2-8) NeuNAc(α2-3) Gal residue in GD1b, GT1b, and GQ1b gangliosides (dotted lines) are shown.

Figure 7 demonstrates that the anti-glycolipid antibody in the blood of patients with mantle cell lymphoma reacted with the peripheral motor nerves. This seemed to be a significant etiological factor^7)^.

Table 3 illustrates our finding that malignant lymphoma itself produces cytokines (TGFbeta1, TNFalpha) and thereby induces fibrosis. This seemed to serve as a significant etiological factor^8)^.

**Table 3:** Evaluation of the odds ratios for fibrosis associated with positive immunostaining regarding TGF-beta1, TNF-alpha1, and combination of TGF and TNF^8)^

	n	Fibrosis vs TGF		Fibrosis vs TNF		Fibrosis vs TGF+TNF	
		Odds Ratio (95%CI)	p-value	Odds Ratio (95%CI)	p-value	Odds Ratio (95%CI)	p-value
All tissue	104	12.80 (4.94-33.20)	< 0.001**	1.75 (0.79-3.90)	0.171	3.35 (1.87-6.00)	< 0.001**
Bone marrows	27	13.70 (2.05-92.00)	0.007**	2.75 (0.55-13.70)	0.218	4.60 (1.31-16.1)	0.017*
AML	7	−	−	−	−	1.00 (0.11-8.75)	1.000
ALL	7	−	−	−	−	−	−
DLBCL	40	12.80 (2.48-66.00)	0.002**	2.04 (0.52-8.00)	0.308	4.45 (1.43-13.80)	0.010*
FL	11	3.50 (0.15-84.70)	0.441	1.25 (0.06-26.90)	0.887	1.54 (0.27-8.69)	0.626
T-cell lymphoma	8	−	−	0.50 (0.02-12.90)	0.676	2.51 (0.23-27.40)	0.451
Others	14	−	−	1.33 (0.14-12.80)	0.803	3.91 (0.79-19.30)	0.094

Notes: CI, confidence interval Odds ratios are relatively accuracy in fibrosis of all disease vs TGF betal, especially in DLBCL.

**Figure 8:**
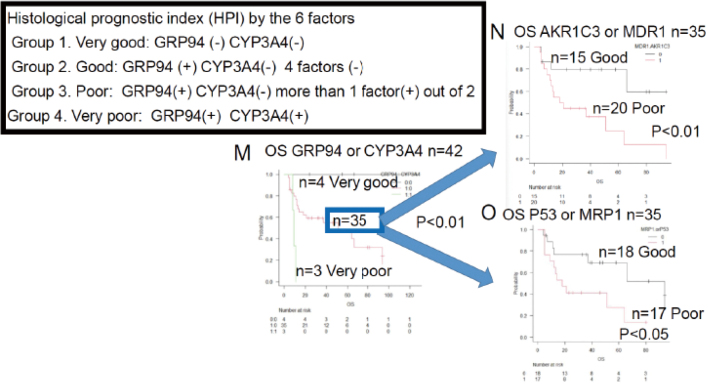
Overall survival of large B-cell lymphoma patients with and without the prognostic factors Kaplan-Meier survival curves and between-group comparisons^9)^ M. GRP94- CYP3A4- n = 4 Group1 Very good (5-year OS 100%) GRP94+ CYP3A4- n = 35 In the Group 4 (Very poor, n = 3) consisting of 3 patients with positivity for both GRP94 and CYP3A4. All the 3 patients died within a short period. p < 0.01 N. AKR1C3- or MDR1- negative n = 15, positive n = 20, p < 0.01 O. P53 or MRP1 negative n = 18, positive n = 17, p < 0.05 Group 1 (n = 4), the “Very good” group, consisted of 4 patients who showed negative staining for both GRP94 and CYP3A4, including 2 patients who were censored. This group had an extremely good prognosis, and all the 4 patients survived (5-year OS: 100%). On the contrary, Group 4 (n = 3), the “very poor” group, consisted of 3 patients who showed positive staining for both GRP94 and CYP3A4. This group had a very poor prognosis and all the 3 patients died within a short period of time. The prognosis of patients in the Group 2 and 3 (n = 35) was intermediate, with the median survival of about 51 months. In Figure 1N and O, the intermediate prognosis group, that is, Group 2 (n = 35), is subdivided into “Group 2 (Good),” consisting of patients who showed negative staining for both AKR1C3 and MDR1, and “Group 2 (Good)” consisting of patients who showed negative staining for both p53 and MRP1. The remaining of Group 3 had a poor prognosis.

Around 2018, I began to study the mechanisms of resistance of hematopoietic tumors to treatment. At present, I am submitting a paper on therapy resistance of large B-cell lymphoma (Figure 8). The new histological prognostic index is important for predicting the responses of large B-cell lymphoma to treatment. Prediction of the treatment response involves 6 factors (GRP94, CYP3A4, AKR1C3, MDR1 and MRP1 P53)^9)^.

Thanks to our Chairman Ogawa, in April 2023, I began to work as a specially appointed professor at the current department (Department of Hematology, Juntendo University Urayasu Hospital). From now on, I plan to work actively to extend cooperation for the attempts of residents and other medical members of our department to make presentations, publish papers, etc., about their research.

Recently, I have begun to feel deeply grateful for Juntendo university's academic motto of “Benevolence,” its principle of “Uninterrupted Advancing,” and its academic position of “three noes principle, *Sanmu* Principle (no discrimination based on gender, nationality, or academic background).” I hope that you will all remain active under these principles and ideas, while taking due care of your own health. I wish to express my appreciation for your continued support and cooperation.

## Funding

No funding was received.

## Author contributions

MN wrote, read and approved the final manuscript.

## Conflicts of interest statement

A Conflict of Interest statement is not included for each of the authors.

Author has no conflicts of interest.
